# (3aR*,6S*,7aR*)-7a-Chloro-6-methyl-2-(4-methyl­phenyl­sulfon­yl)-2,3,3a,6,7,7a-hexa­hydro-3a,6-ep­oxy-1*H*-isoindole

**DOI:** 10.1107/S1600536812009658

**Published:** 2012-03-17

**Authors:** Ersin Temel, Aydın Demircan, Gözde Beyazova, Orhan Büyükgüngör

**Affiliations:** a Ondokuz Mayıs University, Arts and Sciences Faculty, Department of Physics, 55139 Samsun, Turkey; b Nigde University, Faculty of Arts and Sciences, Department of Chemistry, 51240 Nigde, Turkey

## Abstract

In the title compound, C_16_H_18_ClNO_3_S, the six-membered ring has a boat conformation. The two five-membered rings with the bridging O atom adopt envelope conformations, whereas the N-containing five-membered ring adopts a twisted conformation. In the crystal, C—H⋯O hydrogen bonds link the mol­ecules into a three-dimensional network.

## Related literature

For background to the intra­molecular Diels–Alder reaction with furan (IMDAF) as diene partner, see: Lipshutz (1986 ▶); Heiner *et al.* (1996 ▶); Prajapati *et al.* (2000 ▶); Kappe *et al.* (1997 ▶); Padwa *et al.* (1997 ▶). For our studies of the intra­molecular free radical reaction of furan with a carbon side chain, see: Demircan & Parsons (1998 ▶, 2002 ▶); Demircan *et al.* (2006 ▶); Karaarslan *et al.* (2007 ▶). For our investigation of whether the protective group on nitro­gen influences the cyclo­addition process, see: Koşar *et al.* (2006 ▶); Arslan *et al.* (2008 ▶); Temel *et al.* (2011 ▶); Demircan *et al.* (2011 ▶). For puckering analysis, see: Cremer & Pople (1975 ▶). For graph-set notation, see: Bernstein *et al.* (1995 ▶).
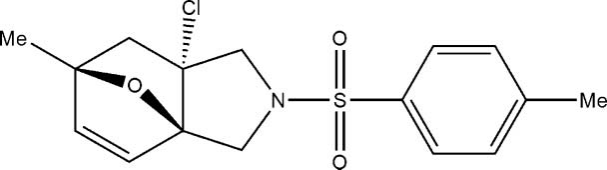


## Experimental

### 

#### Crystal data

C_16_H_18_ClNO_3_S
*M* *_r_* = 339.82
Monoclinic, 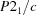

*a* = 10.0523 (5) Å
*b* = 15.5135 (6) Å
*c* = 11.2729 (6) Å
β = 114.800 (4)°
*V* = 1595.84 (13) Å^3^
*Z* = 4
Mo *K*α radiation
μ = 0.38 mm^−1^
*T* = 296 K
0.78 × 0.72 × 0.60 mm

#### Data collection

Stoe IPDS 2 diffractometer
Absorption correction: integration (*X-RED*; Stoe & Cie, 2001 ▶) *T*_min_ = 0.746, *T*_max_ = 0.843
18547 measured reflections
3312 independent reflections
2841 reflections with *I* > 2σ(*I*)
*R*_int_ = 0.026

#### Refinement

*R*[*F* ^2^ > 2σ(*F* ^2^)] = 0.038
*wR*(*F* ^2^) = 0.101
*S* = 1.05
3312 reflections
201 parameters
H-atom parameters constrained
Δρ_max_ = 0.34 e Å^−3^
Δρ_min_ = −0.37 e Å^−3^

### 

Data collection: *X-AREA* (Stoe & Cie, 2002 ▶); cell refinement: *X-AREA*; data reduction: *X-RED32* (Stoe & Cie, 2002 ▶); program(s) used to solve structure: *SHELXS97* (Sheldrick, 2008 ▶); program(s) used to refine structure: *SHELXL97* (Sheldrick, 2008 ▶); molecular graphics: *ORTEP-3 for Windows* (Farrugia, 1997 ▶); software used to prepare material for publication: *WinGX* (Farrugia, 1999 ▶).

## Supplementary Material

Crystal structure: contains datablock(s) I, global. DOI: 10.1107/S1600536812009658/zq2155sup1.cif

Structure factors: contains datablock(s) I. DOI: 10.1107/S1600536812009658/zq2155Isup2.hkl

Supplementary material file. DOI: 10.1107/S1600536812009658/zq2155Isup3.cml

Additional supplementary materials:  crystallographic information; 3D view; checkCIF report

## Figures and Tables

**Table 1:** Hydrogen-bond geometry (Å, °)

*D*—H⋯*A*	*D*—H	H⋯*A*	*D*⋯*A*	*D*—H⋯*A*
C2—H2⋯O1^i^	0.93	2.48	3.368 (2)	159
C5—H5⋯O3^ii^	0.93	2.62	3.539 (2)	169
C13—H13⋯O3^iii^	0.93	2.67	3.601 (2)	174

Symmetry codes: (i) 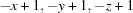; (ii) 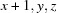; (iii) 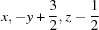.

**Fig. 1.**
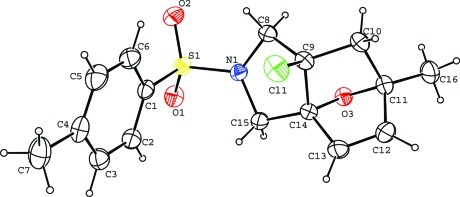
The molecular structure of the title compound, with displacement ellipsoids drawn at the 30% probability level.

**Fig. 2.**
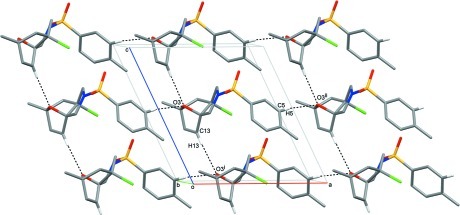
Part of the crystal structure of the title compound, showing the formation of C(4), C(10) chains and R43(25) rings parallel to ac-plane. Hydrogen bonds are indicated by dashed lines. (Symmetry codes: i; x, -y + 3/2, z - 1/2; ii; x + 1, y, z).

**Fig. 3.**
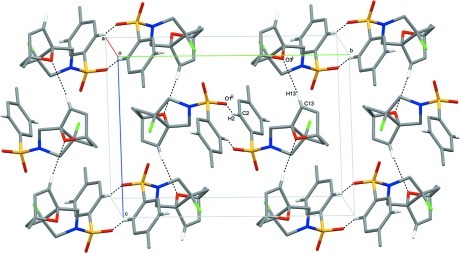
Part of the crystal structure of the title compound, showing the formation of R22(10) and R66(38) rings. Hydrogen bonds are indicated by dashed lines. (Symmetry codes: i; x, -y + 3/2, z - 1/2; ii; -x + 1, -y + 1, -z + 1).

**Fig. 4.**
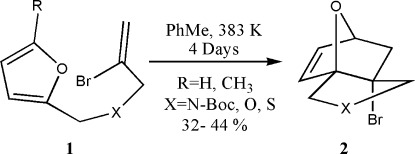
Synthesis of fused tricycles (2) from bromofurufurylalkenes (1)

**Fig. 5.**
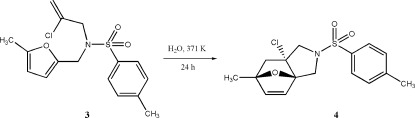
Synthesis of titled compound (4) in water

## supplementary crystallographic information

### Comment

The intramolecular Diels–Alder reaction with furan (IMDAF) as diene partner provides a facile route to the synthesis of complicated multicyclic structures *via* oxanobornenes in synthetic organic chemistry (Lipshutz, 1986). However, in many cases, the cyclization process of IMDAF requires high pressure (Heiner *et al.*, 1996) or the employment of Lewis acid catalysis (Prajapati *et al.*, 2000) to proceed, the most extensively studied five-membered heterocycle for this cycloaddition is furan (Kappe *et al.*, 1997; Padwa *et al.*, 1997).

We have been studying intramolecular free radical reaction of furan with carbon side chain (Demircan & Parsons, 1998; 2002; Demircan *et al.*, 2006; Karaarslan *et al.*, 2007) and recently reported that under thermal conditions the bromofurfurylalkenes (1), with heteroatom possessed in a side chain, undergo intramolecular cycloadditions and give heterofused tricycles (2) (32–44% overall) as shown in Figure 4. We have also been researching whether the protective group on nitrogen influences cycloaddition process or not; it is noteworthy that the exchange of the protective group from *tert*-butoxy (Boc) group to tosyl group increases yield and accelerate the cycloaddition process. We have already reported our findings since 2005 (Koşar *et al.*, 2006; Arslan *et al.*, 2008; Temel *et al.*, 2011; Demircan *et al.*, 2011), now we report the new tricyclic structure, (3aR,6*S*,7aR)-7a-chloro-6-methyl-2-[(4-methylphenyl) sulfonyl]-1,2,3,6,7,7a-hexahydro-3a,6-epoxyisoindole (4), derived from the furan moiety (3) *via* thermal IMDAF in aqueous media with 73% yield (Figure 5).

The molecular structure of the title compound is shown in Figure 1. The title compound contains non-planar five- and six-membered rings. The six membered ring (C9—C14) has a boat conformation with puckering parameters Q = 0.944 (2) Å, θ = 89.03 (12)°, φ = 119.03 (14)°. The two five-membered rings with bridging oxygen (O3/C11/C10/C9/C14 and O3/C11—C14) adopt envelope configurations, whereas the N-containing five membered ring adopts a twisted conformation with the total puckering parameters of 0.6053 (19)°, 0.502 (2)° and 0.324 (2)°, respectively (Cremer & Pople, 1975). The crystal packing is stabilized by intermolecular C—H···O type hydrogen bonds (Table 1). Atom C13 in the reference molecule acts as a hydrogen bond donor to the bridging oxygen atom O3^iii ^forming a C(4) chain running parallel to the *c* axis (*iii* = *x*, -*y* + 3/2, *z* - 1/2). Similarly, atom C5 acts as a hydrogen bond donor to the bridging oxygen atom O3^ii ^forming a C(9) chain running parallel to the *a* axis (*ii* = *x*+1, *y*, *z)*. The intersection of the C(4) and C(9) chains produce *R*_4_^3^(25) rings parallel to the *ac* plane (Fig. 2). The C2—H2···O1^i^ (*i* = -*x* + 1, -*y* + 1, -*z* + 1) hydrogen bond produces dimeric *R*^2^_2_(10) rings while the combination of C2—H2···O1^i^ and C13—H13···O3^iii^ hydrogen bonds generate *R*^6^_6_(38) rings (Fig. 3) (Bernstein *et al.*, 1995).

### Experimental

*N*-(2-chloroprop-2-en-1-yl)-4-methyl-*N*-[(5-methyl-2-furyl)methyl] benzenesulfonamide (3) (1 g, 2.94 mmol) and 50 ml water were placed in a 100 ml two neck flask, equipped with a condenser. The mixture was stirred at 371 K for 24 h and monitored by thin layer chromatography. The reaction mixture was then poured into 50 ml e thyl acetate; aqueous part was further washed with 3x50 ml ethyl acetate. The combined organic phases were washed with 50 ml brine, dried over magnesium sulfate and concentrated under reduced pressure. Subsequently, the residue was subjected to flash column chromatography to afford the title compound (4) as pale yellow crystals, re-crystallized from dichloromethane - hexane(1:4), (0.73 g, 73%).

### Refinement

H atoms were positioned geometrically and treated using a riding model, fixing the bond lengths at 0.96, 0.97 and 0.93 Å for CH_3_, CH_2_ and aromatic CH, respectively. The displacement parameters of the H atoms were constrained with *U*_iso_(H) = 1.2*U*_eq_ (aromatic or methylene C) or 1.5*U*_eq_ (methyl C).

### Crystal data

**Table:**

C_16_H_18_ClNO_3_S	*F*(000) = 712
*M**_r_* = 339.82	*D*_x_ = 1.414 Mg m^−^^3^
Monoclinic, *P*2_1_/*c*	Mo *K*α radiation, λ = 0.71073 Å
Hall symbol: -P 2ybc	Cell parameters from 18547 reflections
*a* = 10.0523 (5) Å	θ = 2.0–28.0°
*b* = 15.5135 (6) Å	µ = 0.38 mm^−^^1^
*c* = 11.2729 (6) Å	*T* = 296 K
β = 114.800 (4)°	Block, colourless
*V* = 1595.84 (13) Å^3^	0.78 × 0.72 × 0.60 mm
*Z* = 4	

### Data collection

**Table:**

Stoe IPDS 2 diffractometer	3312 independent reflections
Radiation source: fine-focus sealed tube	2841 reflections with *I* > 2σ(*I*)
Graphite monochromator	*R*_int_ = 0.026
Detector resolution: 6.67 pixels mm^-1^	θ_max_ = 26.5°, θ_min_ = 2.2°
rotation method scans	*h* = −12→12
Absorption correction: integration (*X-RED*; Stoe & Cie, 2001)	*k* = −19→19
*T*_min_ = 0.746, *T*_max_ = 0.843	*l* = −14→14
18547 measured reflections	

### Refinement

**Table:**

Refinement on *F*^2^	Primary atom site location: structure-invariant direct methods
Least-squares matrix: full	Secondary atom site location: difference Fourier map
*R*[*F*^2^ > 2σ(*F*^2^)] = 0.038	Hydrogen site location: inferred from neighbouring sites
*wR*(*F*^2^) = 0.101	H-atom parameters constrained
*S* = 1.05	*w* = 1/[σ^2^(*F*_o_^2^) + (0.051*P*)^2^ + 0.5399*P*] where *P* = (*F*_o_^2^ + 2*F*_c_^2^)/3
3312 reflections	(Δ/σ)_max_ = 0.001
201 parameters	Δρ_max_ = 0.34 e Å^−^^3^
0 restraints	Δρ_min_ = −0.37 e Å^−^^3^

### Special details

**Table:**

Geometry. All e.s.d.'s (except the e.s.d. in the dihedral angle between two l.s. planes) are estimated using the full covariance matrix. The cell e.s.d.'s are taken into account individually in the estimation of e.s.d.'s in distances, angles and torsion angles; correlations between e.s.d.'s in cell parameters are only used when they are defined by crystal symmetry. An approximate (isotropic) treatment of cell e.s.d.'s is used for estimating e.s.d.'s involving l.s. planes.
Refinement. Refinement of *F*^2^ against ALL reflections. The weighted *R*-factor *wR* and goodness of fit *S* are based on *F*^2^, conventional *R*-factors *R* are based on *F*, with *F* set to zero for negative *F*^2^. The threshold expression of *F*^2^ > σ(*F*^2^) is used only for calculating *R*-factors(gt) *etc*. and is not relevant to the choice of reflections for refinement. *R*-factors based on *F*^2^ are statistically about twice as large as those based on *F*, and *R*- factors based on ALL data will be even larger.

### Fractional atomic coordinates and isotropic or equivalent isotropic displacement parameters (Å^2^)

**Table:**

	*x*	*y*	*z*	*U*_iso_*/*U*_eq_	
C10	0.4256 (2)	0.89806 (12)	0.5996 (2)	0.0456 (4)	
H10A	0.4404	0.9512	0.5614	0.055*	
H10B	0.4544	0.9065	0.6923	0.055*	
C11	0.2649 (2)	0.86575 (12)	0.52936 (19)	0.0454 (4)	
C12	0.2241 (2)	0.86741 (15)	0.3842 (2)	0.0569 (5)	
H12	0.1614	0.9066	0.3244	0.068*	
C13	0.2935 (2)	0.80336 (14)	0.35758 (19)	0.0524 (5)	
H13	0.2893	0.7876	0.2765	0.063*	
C16	0.1574 (3)	0.90085 (17)	0.5776 (3)	0.0670 (6)	
H16A	0.0624	0.8764	0.5276	0.101*	
H16B	0.1522	0.9624	0.5680	0.101*	
H16C	0.1888	0.8862	0.6681	0.101*	
O1	0.61916 (15)	0.52146 (9)	0.65522 (14)	0.0526 (3)	
O2	0.79916 (16)	0.62551 (11)	0.80370 (14)	0.0587 (4)	
O3	0.28849 (13)	0.77360 (8)	0.55435 (12)	0.0413 (3)	
C1	0.77799 (19)	0.60653 (11)	0.56803 (19)	0.0411 (4)	
C2	0.7250 (2)	0.55616 (12)	0.4569 (2)	0.0460 (4)	
H2	0.6428	0.5217	0.4375	0.055*	
C3	0.7952 (2)	0.55757 (13)	0.3748 (2)	0.0520 (5)	
H3	0.7594	0.5236	0.2999	0.062*	
C4	0.9179 (2)	0.60826 (14)	0.4013 (2)	0.0530 (5)	
C5	0.9670 (2)	0.65924 (15)	0.5121 (3)	0.0627 (6)	
H5	1.0481	0.6945	0.5308	0.075*	
C6	0.8985 (2)	0.65894 (14)	0.5953 (2)	0.0562 (5)	
H6	0.9331	0.6937	0.6693	0.067*	
C7	0.9952 (3)	0.60749 (19)	0.3123 (3)	0.0746 (7)	
H7A	1.0581	0.5580	0.3314	0.112*	
H7B	1.0526	0.6590	0.3257	0.112*	
H7C	0.9241	0.6050	0.2230	0.112*	
C8	0.5981 (2)	0.76445 (12)	0.68794 (18)	0.0433 (4)	
H8A	0.7017	0.7778	0.7207	0.052*	
H8B	0.5676	0.7709	0.7585	0.052*	
C9	0.50862 (19)	0.82243 (11)	0.57366 (17)	0.0388 (4)	
C14	0.38083 (19)	0.76143 (11)	0.48755 (16)	0.0377 (4)	
C15	0.4437 (2)	0.67277 (12)	0.5043 (2)	0.0465 (4)	
H15A	0.3726	0.6299	0.5025	0.056*	
H15B	0.4764	0.6595	0.4367	0.056*	
Cl1	0.61872 (6)	0.85248 (4)	0.48951 (6)	0.06283 (18)	
N1	0.56832 (16)	0.67646 (10)	0.63391 (15)	0.0429 (4)	
S1	0.69313 (5)	0.60262 (3)	0.67681 (5)	0.04233 (14)	

### Atomic displacement parameters (Å^2^)

**Table:**

	*U*^11^	*U*^22^	*U*^33^	*U*^12^	*U*^13^	*U*^23^
C10	0.0524 (11)	0.0356 (9)	0.0507 (11)	−0.0016 (8)	0.0233 (9)	−0.0048 (8)
C11	0.0477 (10)	0.0396 (9)	0.0482 (10)	0.0060 (8)	0.0194 (9)	−0.0017 (8)
C12	0.0608 (13)	0.0518 (12)	0.0460 (11)	0.0136 (10)	0.0106 (10)	0.0068 (9)
C13	0.0617 (12)	0.0560 (12)	0.0343 (9)	0.0062 (10)	0.0150 (9)	−0.0004 (8)
C16	0.0531 (12)	0.0716 (15)	0.0790 (16)	0.0095 (11)	0.0304 (12)	−0.0167 (13)
O1	0.0568 (8)	0.0384 (7)	0.0644 (9)	−0.0005 (6)	0.0272 (7)	0.0103 (6)
O2	0.0523 (8)	0.0646 (10)	0.0461 (8)	0.0066 (7)	0.0079 (7)	0.0030 (7)
O3	0.0394 (6)	0.0404 (7)	0.0464 (7)	−0.0014 (5)	0.0202 (6)	0.0000 (5)
C1	0.0347 (9)	0.0351 (9)	0.0498 (10)	0.0034 (7)	0.0141 (8)	0.0043 (8)
C2	0.0425 (9)	0.0377 (10)	0.0560 (11)	−0.0035 (8)	0.0191 (9)	0.0003 (8)
C3	0.0515 (11)	0.0491 (12)	0.0552 (11)	0.0028 (9)	0.0222 (10)	−0.0006 (9)
C4	0.0419 (10)	0.0527 (12)	0.0673 (13)	0.0126 (9)	0.0258 (10)	0.0169 (10)
C5	0.0428 (11)	0.0602 (13)	0.0863 (17)	−0.0092 (10)	0.0282 (12)	0.0013 (12)
C6	0.0413 (10)	0.0536 (12)	0.0699 (14)	−0.0096 (9)	0.0195 (10)	−0.0109 (10)
C7	0.0634 (14)	0.0879 (19)	0.0885 (18)	0.0160 (13)	0.0475 (14)	0.0213 (15)
C8	0.0438 (10)	0.0382 (9)	0.0429 (9)	−0.0027 (7)	0.0132 (8)	−0.0068 (7)
C9	0.0436 (9)	0.0352 (9)	0.0428 (9)	−0.0058 (7)	0.0233 (8)	−0.0024 (7)
C14	0.0386 (9)	0.0379 (9)	0.0357 (9)	−0.0002 (7)	0.0148 (7)	−0.0035 (7)
C15	0.0402 (9)	0.0401 (10)	0.0494 (11)	0.0012 (8)	0.0094 (8)	−0.0076 (8)
Cl1	0.0688 (3)	0.0623 (3)	0.0770 (4)	−0.0109 (3)	0.0498 (3)	0.0005 (3)
N1	0.0385 (8)	0.0383 (8)	0.0441 (8)	0.0008 (6)	0.0097 (7)	−0.0032 (6)
S1	0.0401 (2)	0.0385 (2)	0.0444 (3)	0.00261 (18)	0.01385 (19)	0.00562 (19)

### Geometric parameters (Å, º)

**Table:**

C10—C9	1.537 (2)	C3—C4	1.385 (3)
C10—C11	1.554 (3)	C3—H3	0.9300
C10—H10A	0.9700	C4—C5	1.383 (3)
C10—H10B	0.9700	C4—C7	1.505 (3)
C11—O3	1.457 (2)	C5—C6	1.377 (3)
C11—C16	1.501 (3)	C5—H5	0.9300
C11—C12	1.511 (3)	C6—H6	0.9300
C12—C13	1.319 (3)	C7—H7A	0.9600
C12—H12	0.9300	C7—H7B	0.9600
C13—C14	1.504 (3)	C7—H7C	0.9600
C13—H13	0.9300	C8—N1	1.473 (2)
C16—H16A	0.9600	C8—C9	1.518 (3)
C16—H16B	0.9600	C8—H8A	0.9700
C16—H16C	0.9600	C8—H8B	0.9700
O1—S1	1.4302 (14)	C9—C14	1.563 (2)
O2—S1	1.4245 (15)	C9—Cl1	1.7952 (17)
O3—C14	1.432 (2)	C14—C15	1.492 (2)
C1—C2	1.380 (3)	C15—N1	1.473 (2)
C1—C6	1.382 (3)	C15—H15A	0.9700
C1—S1	1.7643 (19)	C15—H15B	0.9700
C2—C3	1.380 (3)	N1—S1	1.6159 (16)
C2—H2	0.9300		
			
C9—C10—C11	100.86 (14)	C5—C6—H6	120.3
C9—C10—H10A	111.6	C1—C6—H6	120.3
C11—C10—H10A	111.6	C4—C7—H7A	109.5
C9—C10—H10B	111.6	C4—C7—H7B	109.5
C11—C10—H10B	111.6	H7A—C7—H7B	109.5
H10A—C10—H10B	109.4	C4—C7—H7C	109.5
O3—C11—C16	111.69 (17)	H7A—C7—H7C	109.5
O3—C11—C12	100.03 (15)	H7B—C7—H7C	109.5
C16—C11—C12	118.48 (19)	N1—C8—C9	104.64 (14)
O3—C11—C10	99.65 (14)	N1—C8—H8A	110.8
C16—C11—C10	116.82 (17)	C9—C8—H8A	110.8
C12—C11—C10	107.31 (17)	N1—C8—H8B	110.8
C13—C12—C11	107.74 (18)	C9—C8—H8B	110.8
C13—C12—H12	126.1	H8A—C8—H8B	108.9
C11—C12—H12	126.1	C8—C9—C10	117.81 (15)
C12—C13—C14	104.62 (17)	C8—C9—C14	102.03 (13)
C12—C13—H13	127.7	C10—C9—C14	102.13 (14)
C14—C13—H13	127.7	C8—C9—Cl1	108.97 (13)
C11—C16—H16A	109.5	C10—C9—Cl1	113.96 (13)
C11—C16—H16B	109.5	C14—C9—Cl1	110.94 (12)
H16A—C16—H16B	109.5	O3—C14—C15	112.85 (15)
C11—C16—H16C	109.5	O3—C14—C13	102.29 (14)
H16A—C16—H16C	109.5	C15—C14—C13	124.43 (16)
H16B—C16—H16C	109.5	O3—C14—C9	97.98 (12)
C14—O3—C11	96.70 (13)	C15—C14—C9	106.57 (14)
C2—C1—C6	120.26 (19)	C13—C14—C9	109.59 (15)
C2—C1—S1	119.75 (14)	N1—C15—C14	103.22 (14)
C6—C1—S1	119.98 (16)	N1—C15—H15A	111.1
C3—C2—C1	119.22 (18)	C14—C15—H15A	111.1
C3—C2—H2	120.4	N1—C15—H15B	111.1
C1—C2—H2	120.4	C14—C15—H15B	111.1
C2—C3—C4	121.6 (2)	H15A—C15—H15B	109.1
C2—C3—H3	119.2	C15—N1—C8	112.74 (14)
C4—C3—H3	119.2	C15—N1—S1	119.98 (12)
C5—C4—C3	117.9 (2)	C8—N1—S1	122.37 (12)
C5—C4—C7	121.2 (2)	O2—S1—O1	120.46 (9)
C3—C4—C7	120.9 (2)	O2—S1—N1	106.49 (9)
C6—C5—C4	121.5 (2)	O1—S1—N1	106.87 (8)
C6—C5—H5	119.3	O2—S1—C1	108.24 (9)
C4—C5—H5	119.3	O1—S1—C1	106.29 (9)
C5—C6—C1	119.5 (2)	N1—S1—C1	107.98 (8)
			
C9—C10—C11—O3	34.16 (17)	C12—C13—C14—C15	162.03 (19)
C9—C10—C11—C16	154.56 (19)	C12—C13—C14—C9	−70.3 (2)
C9—C10—C11—C12	−69.61 (18)	C8—C9—C14—O3	83.30 (14)
O3—C11—C12—C13	−30.6 (2)	C10—C9—C14—O3	−38.96 (16)
C16—C11—C12—C13	−152.2 (2)	Cl1—C9—C14—O3	−160.76 (11)
C10—C11—C12—C13	72.9 (2)	C8—C9—C14—C15	−33.50 (18)
C11—C12—C13—C14	−0.8 (2)	C10—C9—C14—C15	−155.76 (15)
C16—C11—O3—C14	174.89 (17)	Cl1—C9—C14—C15	82.44 (16)
C12—C11—O3—C14	48.62 (16)	C8—C9—C14—C13	−170.56 (15)
C10—C11—O3—C14	−61.04 (15)	C10—C9—C14—C13	67.19 (17)
C6—C1—C2—C3	1.2 (3)	Cl1—C9—C14—C13	−54.62 (17)
S1—C1—C2—C3	−177.82 (15)	O3—C14—C15—N1	−80.61 (17)
C1—C2—C3—C4	0.0 (3)	C13—C14—C15—N1	154.70 (18)
C2—C3—C4—C5	−1.2 (3)	C9—C14—C15—N1	25.80 (18)
C2—C3—C4—C7	178.6 (2)	C14—C15—N1—C8	−8.4 (2)
C3—C4—C5—C6	1.1 (3)	C14—C15—N1—S1	−164.23 (13)
C7—C4—C5—C6	−178.7 (2)	C9—C8—N1—C15	−12.7 (2)
C4—C5—C6—C1	0.1 (3)	C9—C8—N1—S1	142.44 (13)
C2—C1—C6—C5	−1.3 (3)	C15—N1—S1—O2	178.27 (15)
S1—C1—C6—C5	177.75 (17)	C8—N1—S1—O2	24.88 (17)
N1—C8—C9—C10	137.96 (16)	C15—N1—S1—O1	−51.77 (16)
N1—C8—C9—C14	27.15 (17)	C8—N1—S1—O1	154.84 (15)
N1—C8—C9—Cl1	−90.22 (14)	C15—N1—S1—C1	62.21 (16)
C11—C10—C9—C8	−108.28 (17)	C8—N1—S1—C1	−91.18 (16)
C11—C10—C9—C14	2.47 (17)	C2—C1—S1—O2	153.66 (15)
C11—C10—C9—Cl1	122.17 (14)	C6—C1—S1—O2	−25.36 (19)
C11—O3—C14—C15	173.57 (15)	C2—C1—S1—O1	22.93 (17)
C11—O3—C14—C13	−50.39 (16)	C6—C1—S1—O1	−156.09 (16)
C11—O3—C14—C9	61.76 (14)	C2—C1—S1—N1	−91.44 (16)
C12—C13—C14—O3	32.9 (2)	C6—C1—S1—N1	89.54 (17)

### Hydrogen-bond geometry (Å, º)

**Table:**

*D*—H···*A*	*D*—H	H···*A*	*D*···*A*	*D*—H···*A*
C2—H2···O1^i^	0.93	2.48	3.368 (2)	159
C5—H5···O3^ii^	0.93	2.62	3.539 (2)	169
C13—H13···O3^iii^	0.93	2.67	3.601 (2)	174

Symmetry codes: (i) −*x*+1, −*y*+1, −*z*+1; (ii) *x*+1, *y*, *z*; (iii) *x*, −*y*+3/2, *z*−1/2.
